# Data for a pre-performance test of self-developed electronic tongue sensors

**DOI:** 10.1016/j.dib.2016.11.041

**Published:** 2016-11-19

**Authors:** Laura Isabell Immohr, Roy Turner, Miriam Pein-Hackelbusch

**Affiliations:** a Heinrich-Heine-University Duesseldorf, Institute of Pharmaceutics and Biopharmaceutics, Universitaetsstrasse 1, 40225 Duesseldorf, Germany; b Novartis Pharma AG, Technical Research & Development, 4056 Basel, Switzerland; c University of Applied Sciences Ostwestfalen-Lippe, Life Sciences Technologies, Georg-Weerth-Strasse 20, 32756 Detmold, Germany

**Keywords:** e-tongue, Performance testing, Conditioning cycles

## Abstract

This article presents data, which can be applied for a pre-performance test of self-developed electronic tongue sensors. Contained data is related to the research article “Impact of Sodium Lauryl Sulfate in oral liquids on E-Tongue Measurements” (http://dx.doi.org/10.1016/j.ijpharm.2016.10.045; (L.I. Immohr, R. Turner, M. Pein-Hackelbusch, 2016) [1]). Sensor responses were obtained from 10 subsequent measurements and four different concentrations of quinine hydrochloride by electronic tongue (TS-5000Z, Insent Inc., Atsugi-Shi, Japan) measurements. Based on the data for the pre-performance testing, which were calculated based on the fluctuation range of the sensor responses around the median, stability criteria and required preconditions cycles were defined.

**Specifications Table**

**Table:** 

Subject area	*Chemistry*
More specific subject area	*Pharmaceutical analysis*
Type of data	*Figure*
How data was acquired	*Electronic tongue measurement (sensor potentials)*
Data format	*Relative sensor potentials*
Experimental factors	*Absolute sensor responses were corrected by an internal standard, sensor responses of the first out of five measurement runs were discarded.*
Experimental features	*Self-developed membrane electrodes were produced by solvent casting and their suitability to give a stable and concentration dependent sensor response to quinine hydrochloride dihydrate was evaluated.*
Data source location	*Duesseldorf, Germany; Basel, Switzerland*
Data accessibility	*Data are presented in this article.*

**Value of the data**

• A method to test the performance of self-developed electronic tongue sensors prior to their use in pharmaceutical analysis is suggested.
• Data of the brief pre-testing could be applied to sort unsuitable and damaged sensors.
• Dependent on the purpose of the planned study, data of the performance test help ensuring the sensitivity of the sensors towards a sample molecule.
• Data helps identifying the preconditioning cycles.

## 1 Data

**Fig. 1:**
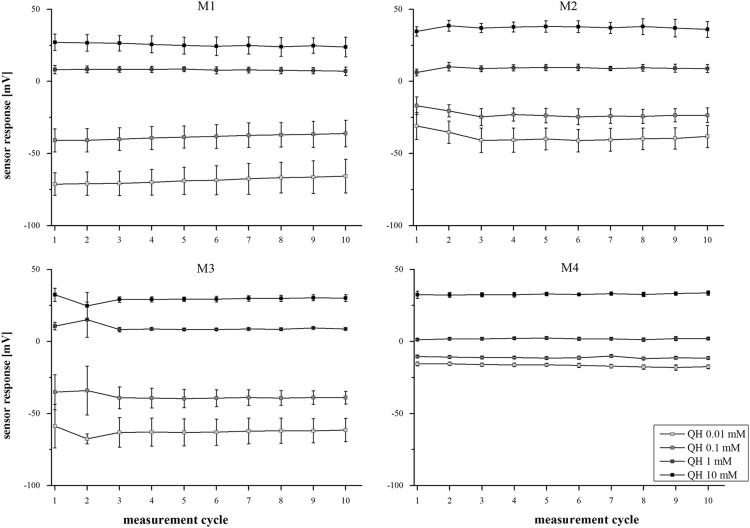
Sensor performance of the self-developed sensors tested prior to the execution the subsequent experiments [1] over 10 measurements cycles (*n*=4 sensors, mean±s).

Data presented in this article (Fig. 1) enable an objective assessment of the performance of (self-developed) e-tongue sensors by calculation of the fluctuation range of the sensor responses around the median (Table 1) as surrogate for the stability of the sensor responses.

## 2 Experimental design, materials and methods

### 2.1 Samples and measurements

Performance of self-developed sensors was evaluated with regard to their sensitivity towards the bitter model drug quinine hydrochloride dihydrate. The drug compound was analyzed in four concentrations (0.01 mM, 0.1 mM, 1 mM and 10 mM) over 10 measurements cycles to ensure a concentration dependent sensor response and a reproducible sensor signal.

### 2.2 Content of self-developed sensor membranes

Prepared sensor membranes (M1–M4) contained PVC, isopropylmyristate (M1–M3), 2-nitro-phenyl octyl ether (M4), hydroxyl-propyl-ß-cyclodextrin (M2 and M4), a cyclodextrin oligomer (M1 and M3), trioctylmethyl ammonium chloride, Bis(2-ethylhexyl) phosphate (M1 and M3) and/or oleic acid (M1 and M3-M4). E-tongue sensors were then prepared according to [1].

### 2.3 Data evaluation by e-tongue measurements

Sensor responses of *n=*4 sensors of each type (M1–M4) were evaluated by electronic tongue measurements (Insent taste sensing system TS-5000Z). Measurement protocol was used according to [2] with a stability criterion of 2 mV.

Sensor responses stabilized after a maximum of three measurement cycles, representing the preconditioning step of the sensors (Fig. 1). All sensors used in the related study [1], show a stable sensor response over a minimum of seven measurement cycles and a concentration dependent signal towards the model drug compound quinine hydrochloride dihydrate. The stability of the sensor signals can be defined by the intervals between the minimum and maximum sensor response of the four sensors of one type (Table 1). These intervals depict the range in which the sensor responses fluctuate. Dependent from the absolute sensor potential a range of <10 mV (a variation of 5 mV around the median) was defined as a stable signal. Accordingly, data for sensors M1, M3 and M4 proved unrestricted stable signals, whereas sensor M2 showed a slight deviation for 0.01 mM quinine hydrochloride dihydrate. This variance is only covered by the first three measurements (Fig. 1) and according data can be used to define the preconditioning phase. Fluctuation for a minimum of seven measurements was sufficient to comply with the stability criterion for the self-developed sensors.

## Figures and Tables

**Table 1:** Intervals between the minimum and maximum sensor responses out of 10 measurement cycles at the 4 different quinine hydrochloride dihydrate (QH) samples as an indicator for the signal stability of the sensors (in mV, *n*=4 sensors/measurement).

	M1	M2	M3	M4
QH 0.01 mM	5.5	10.1	8.8	2.6
QH 0.1 mM	4.8	7.8	5.5	1.7
QH 1 mM	1.4	4.0	6.9	1.2
QH 10 mM	3.3	4.0	7.7	1.3
